# Coexistence of Primary Hyperaldosteronism and Graves' Disease, a Rare Combination of Endocrine Disorders: Is It beyond a Coincidence—A Case Report and Review of the Literature

**DOI:** 10.1155/2017/4050458

**Published:** 2017-10-30

**Authors:** S. S. C. Gunatilake, U. Bulugahapitiya

**Affiliations:** Department of Endocrinology, Colombo South Teaching Hospital, Kalubowila, Sri Lanka

## Abstract

**Background:**

Primary hyperaldosteronism is a known cause for secondary hypertension. In addition to its effect on blood pressure, aldosterone exhibits proinflammatory actions and plays a role in immunomodulation/development of autoimmunity. Recent researches also suggest significant thyroid dysfunction among patients with hyperaldosteronism, but exact causal relationship is not established. Autoimmune hyperthyroidism (Graves' disease) and primary hyperaldosteronism rarely coexist but underlying mechanisms associating the two are still unclear.

**Case Presentation:**

A 32-year-old Sri Lankan female was evaluated for new onset hypertension in association with hypokalemia. She also had features of hyperthyroidism together with high TSH receptor antibodies suggestive of Graves' disease. On evaluation of persistent hypokalemia and hypertension, primary hyperaldosteronism due to right-sided adrenal adenoma was diagnosed. She was rendered euthyroid with antithyroid drugs followed by right-sided adrenalectomy. Antithyroid drugs were continued up to 12 months, after which the patient entered remission of Graves' disease.

**Conclusion:**

Autoimmune hyperthyroidism and primary hyperaldosteronism rarely coexist and this case report adds to the limited number of cases documented in the literature. Underlying mechanism associating the two is still unclear but possibilities of autoimmune mechanisms and autoantibodies warrant further evaluation and research.

## 1. Background

Primary hyperaldosteronism (PA) is a leading endocrine cause for secondary hypertension, particularly in resistant hypertension [1, 2]. In addition to the hypertensive effect by aldosterone, it also exhibits proinflammatory actions on different organ systems, particularly cardiovascular system [3, 4]. Recent studies have demonstrated role of aldosterone on immunomodulation together with its effects on adaptive immune system, suggesting the possible link with development of autoimmune disorders [5]. Graves' disease is an autoimmune disease involving the thyroid gland resulting in thyrotoxicosis secondary to thyroid receptor autoantibodies. It accounts for up to 60–80% of all causes of thyrotoxicosis worldwide [6, 7]. There is a paucity of literature detailing any association between PA and Graves' disease. We report a case of PA due to adrenocortical adenoma (Conn's syndrome) coexisting with Graves' disease in the same patient and review the available literature in view of identifying possible associations.

## 2. Case Presentation

A 32-year-old Sri Lankan female was referred to endocrine unit for further evaluation and management of hypertension and hypokalemia.

She was diagnosed to have hypertension while evaluating for persistent headache 4 months before. She was on three antihypertensive medications at the time of presentation but had poor blood pressure control. She also had nonspecific body aches and intermittent muscle cramps for the past 2 months following which a biochemical evaluation revealed persistent hypokalemia. She also had palpitation and sweating with associated heat intolerance, recent weight loss, and increased bowel openings. She did not have any virilizing features. She was not on diuretics or any long-term medications except three antihypertensive medications. None of her immediate family members had hypertension, strokes, or sudden deaths at younger age.

On examination, she was averagely built with a BMI of 23 kg/m^2^. There was a small diffusely enlarged goiter (grade II) without any tenderness. A bruit was audible over the goiter and no cervical lymphadenopathy was detected. Her eyes were normal including eye movements and vision. There were fine tremors in the fingers with sweaty palms. No characteristic features of Cushing's syndrome were identified. Peripheral pulses were normal without a radio femoral delay. Her pulse rate was 110 beats/minute and blood pressure was 140/100 mmHg while on antihypertensive therapy, without a postural drop. There was no cardiomegaly or any murmurs. Abdominal examination revealed no ballotable masses or renal bruits.

**Figure 1:**
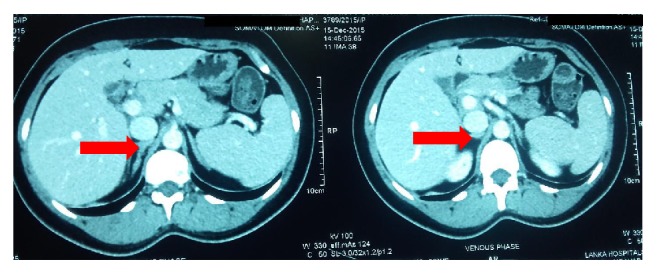
Contrast enhanced CT abdomen showing right-sided adrenal adenoma (red arrow).

Investigations revealed serum potassium, 2.1 mmol/L (3.5–5); spot urinary K, 46 mmol/L (normal < 20); and an arterial pH, 7.48 with bicarbonate of 28 mEq/L. Her serum magnesium level was 1.6 mg/dL (1.7–2.2 mg/dL). In the background of unremarkable physical findings in a patient with hypertension, hypokalemia, high urine potassium excretion, and metabolic alkalosis, possibility of primary hyperaldosteronism was considered. Aldosterone : renin ratio (ARR) was measured after correcting the potassium value and adjusting the interfering medications. Plasma renin activity was 0.15 ng/mL/hr (1.31–3.95) with serum aldosterone 20.6 ng/dL (1–16). ARR was 137 [ng/dL]/[ng/ml/hr] (<20) which is very high, suggesting PA. Intravenous saline infusion test as the test for confirmation of PA revealed basal plasma aldosterone level, 14.30 ng/dL, and postsaline loaded aldosterone level, 13.80 ng/dL (<10). Nonsuppressed aldosterone levels confirmed PA. A contrast enhanced computed tomography (CT) of abdomen according to the adrenal protocol showed a right-sided homogenously dense (density of 9.5 Hounsfield Units {HU}) adrenal lesion measuring 1.6 × 1.3 × 0.8 cm with an absolute washout of 67% confirming benign nature of the lipid rich adrenal adenoma (Figure 1).

Evaluation of the thyroid status revealed evidence of hyperthyroidism: TSH < 0.01 *μ*IU/mL (0.27–4.0), free T4—4.60 ng/dL (0.7–1.9), and free T3—7.36 pg/mL (2–4.4). Ultrasound scan of the thyroid showed diffusely enlarged gland with increased echo pattern and vascularity on Doppler studies, compatible with Graves' disease. TSH receptor antibodies were positive (52 U/L).

Her renal function tests were normal. In addition, she had suppressed cortisol on overnight dexamethasone suppression test and normal 24-hour urinary vanillyl mandelic acid levels on two occasions.

Presence of hyperaldosteronism together with a right-sided adrenal adenoma was consistent with Conn's syndrome in this 32-year-old lady. In addition, there was evidence of coexisting Graves' thyrotoxicosis. Thyrotoxicosis was managed with antithyroid drugs (Carbimazole) according to the titration regimen and once patient rendered euthyroid while on carbimazole, laparoscopic right adrenalectomy was performed. Following the surgery, potassium was 4.4 mmol/l and blood pressure was 130/80 mmHg without medication. Postoperative aldosterone done 3 weeks after the surgery was normal (aldosterone, 4.40 ng/dL), confirming the correct diagnosis of aldosterone secreting adenoma and complete removal of the tumor. Histology revealed adrenocortical adenoma. Antithyroid medications were titrated and discontinued after 12 months following which patient achieved remission of Graves' disease.

## 3. Discussion and Review of Literature

Primary aldosteronism is now considered one of the common causes of secondary hypertension. Cross-sectional and prospective studies report PA in >5% and possibly >10% of hypertensive patients, both in general and in specialty settings [1, 2, 8, 9]. It is an important diagnosis as PA has higher cardiovascular morbidity and mortality than age- and sex-matched patients with essential hypertension with the same degree of blood pressure elevation [10]. These effects may be mediated at least in part by mineralocorticoid receptors in the heart and blood vessels which will enhance impaired endothelial function via reduced glucose 6-phosphate dehydrogenase activity [11, 12].

Aldosterone, in addition to its hypertensive effect, has shown to result in cardiovascular disease by promoting an inflammatory state that is enhanced by T cell immunity, thus establishing its role in the immune system. Aldosterone promotes inflammation characterized by vascular infiltration of immune cells, proinflammatory cytokine production (e.g., TNF *α*), and reactive oxidative stress. Further, it can promote CD4^+^ T cell activation and Th17 polarization suggesting that it plays a role in the adaptive immune system and could contribute to the onset of autoimmunity [3–5, 13]. Herrada et al. had observed aldosterone enhancing the occurrence of autoimmune encephalomyelitis in mice studies, giving further proof. Molina-Garrido et al. had reported a case of primary hyperaldosteronism in whom vitiligo vulgaris and symptomless autoimmune hypothyroidism were identified [14]. Further, proinflammatory actions induced by aldosterone contributing to chronic inflammatory autoimmune diseases was elaborated and reported by Suh et al., describing coexistence of primary aldosteronism with ankylosing spondylitis in a 59-year-old female [15]. This is also supported by the observations by Bendtzen et al. [16], where spironolactone (aldosterone receptor antagonist) reduced tumor necrosis factor-*α* and interferon-*γ* production in patients with rheumatoid and juvenile idiopathic arthritis, thus reducing the inflammation. Therefore, aldosterone may play a significant role in development and progression of autoimmune diseases.

There is emerging evidence of stimulating autoantibodies against angiotensin II type I receptor (AT-1R) which had been isolated from patients with PA [17]. Kem et al. found the prevalence of such antibodies is 31% in patients with PA [18]. 92% of the patients with hyperaldosteronism secondary to primary adrenal adenoma had autoantibodies against AT-1R in one study [19], whereas Li et al. demonstrated a 46% prevalence [20]. AT-1R antibodies may chronically stimulate the zona glomerulosa resulting in hyperproliferative state, which can lead to somatic gain of function mutation, leading to aldosterone producing adenomas. Bilateral adrenal hyperplasia also showed AT-1R antibody positivity in 75% of the patients [20]. These findings support an underlying autoantibody medicated mechanism for PA.

Thyroid dysfunction is a common endocrine disease worldwide. Apart from the direct manifestations due to changes in the thyroid hormone levels, it is associated with several cardiovascular effects. TSH level is found to positively correlate with lipid abnormalities, atherosclerotic disease, diastolic hypertension, and endothelial dysfunction in hypothyroid patients [21]. Suppressed TSH levels are correlated with hypertension, atrial fibrillation, endothelial dysfunction, myocardial infarction, and heart failure in patients with thyrotoxicosis [22]. Graves' thyrotoxicosis is the commonest among all causes of spontaneous thyrotoxicosis. Although B and T lymphocyte-mediated autoimmunity are known to be directed at different antigens in Graves' disease, TSH receptor appears the primary antigenic site, resulting in hyperthyroidism. Main pathogenic mechanism is through stimulatory TSH receptor autoantibodies (TRAb) causing endogenous overproduction of thyroxin hormone.

Although concurrent presence of hyperaldosteronism and thyroid disorders could be a chance occurrence due to relatively high prevalence, presence of a direct association had been discussed for several decades although exact causal relationship is not yet established. A study conducted by Armanini et al. [23] looked into the thyroid abnormalities among 40 patients with PA, secondary to both idiopathic bilateral adrenal hyperplasia (IHA) and unilateral adrenocortical adenoma. It showed that ultrasonographic thyroid abnormalities were present in 60% of the patients with PA compared to 27% in normal controls (*p* < 0.0001). Prevalence of multinodular goiter was significantly higher compared to the controls in the same cohort. In a study done on 188 patients with PA and hypertension, Turchi et al. demonstrated that higher prevalence of ultrasonographic alterations in patients with PA compared to patients with essential hypertension (66% versus 46%, *p* < 0.05) without any significant difference in thyroid function tests [24]. High prevalence of thyroid dysfunction was also observed by Santori et al. among patients with PA {28.6% of patients with PA compared to 16% in patients with essential hypertension (chi^2^ = 0.012)} [25]. The above observations have led to the hypothesis of a common pathogenic mechanisms such as imbalance/interplay between various growth factors and/or inflammatory cytokines, although exact mechanism is not yet identified.

Considering the association of aldosterone with autoimmune disease development, spectrum of autoimmune thyroid disorders could also be considered as associations although a definitive autoimmune syndrome or a cluster is not described. Tanaka et al. had described a 43-year-old lady with combined PA and Cushing's syndrome complicated with Hashimoto' thyroiditis [26]. Krysiak and Okopien [27] described a 36-year-old lady in whom there was primary aldosteronism due to left-sided adrenal adenoma which exacerbated the course of autoimmune thyroid disease (Hashimoto's thyroiditis). The same authors have also noted the elevated proinflammatory cytokines (TNF *α*, interleukin 2, and interferon-*γ*) have reduced after adrenalectomy, which in turn resulted in improvement of thyroid functions and reduction in thyroid autoimmunity in Hashimoto's thyroiditis. An observational study by Sabbadin et al. [28] revealed that the prevalence of anti-thyroid antibodies was significantly higher in PA than in controls (31.5% versus 7.8%) and greater in PA due to adrenal adenomas than IHA (33.3% versus 29.4%). These studies clearly demonstrate the association of aldosterone and thyroid autoimmunity.

Literature on association between PA and thyrotoxicosis is limited. Medline search since 1960 using the terms “primary hyperaldosteronism”, “bilateral adrenal hyperplasia”, “Conn's syndrome”, “hyperthyroidism”, “thyrotoxicosis” “Graves' disease” and “goiter” was performed. Only seven case reports with a title suggesting an association between primary hyperaldosteronism and thyrotoxicosis were found [29–35], but of which no abstract was available in three of the articles (Table 1). Larouche et al. had described a 29-year-old lady in whom there was coexisting primary hyperaldosteronism due to the fact that IHA and Graves' disease were diagnosed, highlighting that PA may be associated with autoimmune hyperthyroidism [32]. Anaforoğlu et al. describe a 51-year-old lady who presented with hypokalemic paralysis precipitated by coexisting left-sided aldosterone secreting adenoma and toxic nodular goiter [33], while Yokota et al. reported a case of PA due to left-sided adrenal adenoma coexisting with Graves' thyrotoxicosis resulting in hypokalemic paralysis, highlighting rarity of the association and importance of diagnosis [34]. A 43-year-old lady with hypokalemia paralysis precipitated by underlying hyperaldosteronism due to left-sided Conn's adenoma and hyperthyroidism was described by Kuo et al. [35] emphasizing the mutual interaction between PA and thyrotoxicosis giving rise to fatal clinical manifestations. Yet, the findings of involvement of stimulatory autoantibodies in both Graves' disease (TRAb) and PA (antibodies against AT-1R) as elaborated earlier in the discussion point out to a definitive autoimmune association in both conditions.

In addition to above observations of coexistence of PA and thyrotoxicosis, thyrotoxicosis itself is a trigger for secondary hyperaldosteronism. Numerous researches have evaluated the state of aldosterone levels in patients with hyperthyroidism and effects of thyroid hormones on synthesis and secretion of renin-angiotensin system [36–40]. They have found an increase in renin and aldosterone levels in thyrotoxic patients compared to euthyroid and hypothyroid patients. This led to the finding of regulation of aldosterone secretion by thyrotoxic state, probably in the form of secondary hyperaldosteronism. Suggested mechanisms are intensified beta-adrenergic activity of the nervous system and increased catecholamine production participated in increase in the mineralocorticoid function of the adrenal glands during thyrotoxicosis and upregulation of renin-angiotensin-aldosterone system [41]. This again highlights the close relationship between the two hormones, thyroxin and aldosterone.

The index case in the current case report gives further evidence of simultaneous presence of Conn's syndrome resulting in PA and Graves' thyrotoxicosis. Although TRAb was performed, AR-1R was not performed due to unavailability. Arterial venous sampling of the adrenal lesions for lateralization was not performed in our patient and surgery was considered directly according to the current available guidelines as it was a small lesion (<2 cm) and patient was under the age of 40 years. Normalization of blood pressure and ARR after the surgery further confirms that PA is due to the adrenal adenoma. Patient had achieved remission of thyrotoxicosis in 12 months after the onset of Graves' disease, much earlier than usually the expected 18-month period. Whether correction of hyperaldosteronism has contributed to the early remission is not clear. Degree of hyperaldosteronism altering the course of autoimmune disease as previously shown by Krysiak and Okopien in relation to Hashimoto's thyroiditis and its application in the course of Graves' disease needs further research.

## 4. Conclusion

In the background of paucity of cases detailing association between the PA and autoimmune thyroid disease, especially Graves' disease, index case illustrates the coexistence of PA in the form of Conn's adenoma and Graves' disease, adding to the limited number of cases described in the literature. It is not certain if the association is incidental, yet the clear association of hyperaldosteronism with autoimmune diseases and detection of stimulatory autoantibodies warrants further evaluation/research for the causal relationship and effect on the progression of the disease and associations, which would in turn aid in the evaluation and management of such autoimmune diseases.

## Figures and Tables

**Table 1:** Reported cases on hyperthyroidism and hyperaldosteronism.

Authors	Year	Patient	Presentation	Thyroid status	Hyperaldosteronism	Other associations
Bru et al.	1963	N/A	N/A	Hyperthyroidism	Hyperaldosteronism	—

Kijima and Sasaoka	1983	N/A	Hypokalemic paralysis	Hyperthyroidism	IHA	—

Iacovlev et al.	1994	N/A	N/A	Hyperthyroidism due to diffuse toxic goiter? Graves' disease	Conn's adenoma	—

Larouche et al.	2015	29 y old female	Psychosis following radioactive therapy for Graves' thyrotoxicosis	Graves' disease	IHA	—

Anaforoğlu et al.	2009	51 y old female	Hypokalemic paralysis	Toxic nodular goiter	Conn's adenoma	Subclinical Cushing's syndrome

Yokota et al.	1991	35 y old male	Hypokalemic paralysis	Graves' disease	Conn's adenoma	—

Kuo et al.	2009	43 y old female	Hypokalemic paralysis	Hyperthyroidism (exact cause not documented)	Conn's adenoma	—

## Abbreviations

ARR: Aldosterone renin ratio
AT-1R: Angiotensin II type I receptor
BMI: Body mass index
CT: Computed tomogram
IHA: Idiopathic hyperaldosteronism
HU: Hounsfield Units
PA: Primary hyperaldosteronism
TRAb: Thyroid stimulating hormone receptor antibodies
TNF *α*: Tumor necrosis factor alpha
TSH: Thyroid stimulating hormone.
